# Thermodynamically consistent model calibration in chemical kinetics

**DOI:** 10.1186/1752-0509-5-64

**Published:** 2011-05-06

**Authors:** Garrett Jenkinson, John Goutsias

**Affiliations:** 1 Whitaker Biomedical Engineering Institute, The Johns Hopkins University, Baltimore, MD 21218, USA

## Abstract

**Background:**

The dynamics of biochemical reaction systems are constrained by the fundamental laws of thermodynamics, which impose well-defined relationships among the reaction rate constants characterizing these systems. Constructing biochemical reaction systems from experimental observations often leads to parameter values that do not satisfy the necessary thermodynamic constraints. This can result in models that are not physically realizable and may lead to inaccurate, or even erroneous, descriptions of cellular function.

**Results:**

We introduce a thermodynamically consistent model calibration (TCMC) method that can be effectively used to provide thermodynamically feasible values for the parameters of an *open *biochemical reaction system. The proposed method formulates the model calibration problem as a constrained optimization problem that takes thermodynamic constraints (and, if desired, additional non-thermodynamic constraints) into account. By calculating thermodynamically feasible values for the kinetic parameters of a well-known model of the EGF/ERK signaling cascade, we demonstrate the qualitative and quantitative significance of imposing thermodynamic constraints on these parameters and the effectiveness of our method for accomplishing this important task. MATLAB software, using the Systems Biology Toolbox 2.1, can be accessed from http://www.cis.jhu.edu/~goutsias/CSS lab/software.html. An SBML file containing the thermodynamically feasible EGF/ERK signaling cascade model can be found in the BioModels database.

**Conclusions:**

TCMC is a simple and flexible method for obtaining physically plausible values for the kinetic parameters of open biochemical reaction systems. It can be effectively used to recalculate a thermodynamically consistent set of parameter values for existing thermodynamically infeasible biochemical reaction models of cellular function as well as to estimate thermodynamically feasible values for the parameters of new models. Furthermore, TCMC can provide dimensionality reduction, better estimation performance, and lower computational complexity, and can help to alleviate the problem of data overfitting.

## Background

Physical systems are constrained to operate according to the fundamental laws of thermodynamics. The conservation of mass and energy and the production of entropy (or heat dissipation) dictate that certain events are physically impossible. A broken glass, for example, will not spontaneously reassemble, and a bar of gold will not fortuitously appear from thin air. Not all physical constraints imposed by thermodynamics are intuitively obvious. As a matter of fact, thermodynamic constraints imposed on biochemical reaction systems are routinely overlooked in the literature, either due to ignorance of their existence or difficulties in understanding the implications of modern non-equilibrium thermodynamics. There is an increasing consensus, however, that care must be taken to ensure that the kinetic parameters of a biochemical reaction system meet these thermodynamic constraints [1-6].

There are many publications discussing the problem of estimating the kinetic parameters of a biochemical reaction system from experimental data of molecular concentrations, when the underlying stoichiometry is known [7-9]. Essentially, all approaches to this problem, which is often referred to as *model calibration*, are based on deriving a cost function and choosing an optimization algorithm to minimize that function [10]. The cost function provides a measure of 'goodness of fit' of the estimated biochemical reaction system dynamics to available observations, and may be designed by a variety of statistical inference techniques, such as maximum likelihood, Bayesian inference, etc., or by simply employing an appropriate distance metric, such as least-squares. The optimization procedure used must be characterized by superior performance for finding global minima, due to the highly non-convex and multi-modal nature of the cost function [10]. The vast majority of published model calibration methods, however, produce biochemical reaction systems that may not be physically realizable, since they do not take into account the fact that the underlying kinetic parameters may be constrained by the fundamental laws of thermodynamics.

Recently, there have been several attempts to address the issue of thermodynamic constraints in chemical kinetics [2-5]. Among proposed methods, the thermodynamic-kinetic modeling (TKM) approach [5] enjoys some benefits over other techniques. However, we have previously noted in [11] that this approach is unnecessarily complicated and can be cumbersome, especially when dealing with molecular perturbations (commonly used in systems biology) or when merging estimated TKM models [5].

To address these problems, we have recently proposed two techniques for estimating the kinetic parameters of *closed *biochemical reaction systems from available observations of molecular concentrations in a thermodynamically consistent fashion [11,12]. In [12], we model biochemical reaction systems by mass-action kinetics, use maximum-likelihood estimation, and employ a projection step that allows us to appropriately choose kinetic parameter values so that the final system is thermodynamically feasible. In [11], we employ a Bayesian inference approach, eliminate the projection step, and derive a biophysically based cost function over parameters that can be chosen independently without violating the underlying thermodynamic constraints. Unfortunately, both methods are limited, being applied to closed biochemical reaction systems using standard mass action kinetics.

In this paper, we propose a method for calibrating the kinetic parameters of biochemical reaction models of cellular function so that the resulting systems are thermodynamically feasible. The method, which we refer to as *Thermodynamically Consistent Model Calibration *(TCMC), works with any iterative parameter estimation algorithm of choice and can be applied to *open *biochemical reaction systems, which, in problems of systems biology, are more realistic than the closed systems we considered previously. Furthermore, TCMC is capable of handling any kinetic rate laws in ideal mixtures, such as non-mass action rate laws commonly used to describe complex enzymatic reaction schemes.

We exemplify practical aspects of the proposed technique by recalculating the kinetic parameters of a well-known model of the EGF/ERK signaling cascade [13], which is thermodynamically infeasible. This allows us to propose a thermodynamically feasible model for this important signaling pathway that is physically realizable and better matches available densitometric data. Computer simulations reveal a number of qualitative and quantitative differences of possible biological significance between the thermodynamically feasible and the thermodynamically infeasible published model, which need to be validated experimentally. Moreover, we discuss a number of important advantages gained by TCMC over estimating the kinetic parameters using a collective fitting approach that does not consider the underlying thermodynamic constraints. Besides producing physically realizable and thermodynamically consistent models, TCMC may result in dimensionality reduction, better estimation performance, and lower computational complexity. MATLAB software, using the Systems Biology Toolbox 2.1 http://www.sbtoolbox2.org, can be accessed from http://www.cis.jhu.edu/~goutsias/CSSlab/software.html. An SBML file containing the thermodynamically feasible EGF/ERK signaling cascade model can be found in the BioModels database http://www.ebi.ac.uk/biomodels-main.

We believe that TCMC can be effectively used to recalculate the parameter values of *any *existing thermodynamically infeasible biochemical reaction model of cellular function, as well as to estimate the parameters of new biochemical reaction models from available experimental data, thus producing physically plausible versions of these models compatible with the fundamental laws of thermodynamics. Finally, as more chemical species or reactions are discovered, TCMC can be used to easily extend existing models of cellular activity in a thermodynamically consistent and computationally efficient fashion.

## Results

### Biochemical Reaction Systems

Most biological processes of interest to systems biology are modeled by means of *open *biochemical reaction systems; i.e., systems that exchange mass with their surroundings [14]. Living cells, for example, are open systems maintaining themselves by exchanging materials with their environment. Mass exchange is modeled either explicitly or implicitly. Examples of explicit modeling include: clamped species, reactions with null species as reactants or products, and irreversible reactions [15]. Clamped species are chemicals whose concentrations are held fixed. They are often used to model molecular species whose concentrations are affected by unknown reactions. It is apparent that these chemicals must be supplied or removed from the system at appropriate rates to ensure that their concentrations do not deviate from their fixed values. On the other hand, reactions with null reactants or products model mass transfer in and out of the system, respectively. Finally, the use of an irreversible reaction is based on the assumption that the concentration of at least one of its products is clamped to zero (otherwise, the reaction can be reversed at sufficiently high product concentrations), which implies mass transfer as well. An example of implicitly modeling mass exchange is a reaction with reactants or products not modeled by the system. A common case would be the phosphorylation of a protein without explicitly modeling conversion of ATP to ADP [5].

An open biochemical reaction system is comprised of *N *molecular species *X*_1_, *X*_2_,..., *X_N _*that interact through *M *coupled reactions, given by

where , ℳ: = {1, 2,..., *M*}, and *ν*_*nm*_,  are the stoichiometries of the reactants and products. We can characterize an open biochemical reaction system at time *t *≥ 0 by the concentrations  of all molecular species at *t*. Clamped molecular species  have concentrations that do not vary with time, whereas, the concentrations of the remaining 'dynamic' species  evolve as a function of time. We will assume that the system characterizes reactions in an ideal and well-stirred (homogeneous) mixture at constant temperature and volume and that the concentrations  vary continuously in time. In this case, we can describe the dynamic evolution of the molecular concentrations in the system by the following chemical kinetic equations:(1)

initialized by setting *x*_*n*_(0) = *q*_*n*_, , for some initial concentrations *q*_*n*_, , where *ϕ_m_*(*t*, **k**) is the net flux of the *m*^th ^reaction,  is the net stoichiometry coefficient of the *n*^th ^molecular species associated with the *m*^th ^reaction,  is an observation time window of interest, and **k **is a vector of kinetic parameters , where . The parameters **k **characterize the biochemical reaction system at hand and are independent of the molecular concentrations . Moreover, we assume that these parameters do not vary with time.

By appropriately pruning and modifying an open biochemical reaction system, we can derive a *closed *subsystem (i.e., a system that does not exchange mass with its surroundings) that lends itself to thermodynamic analysis since external or unknown thermodynamic forces no longer exist. To do so, we first remove all null and irreversible reactions, as well as partially modeled reactions (i.e., reactions with incomplete stoichiometries). Next, we remove clamping of molecular species involved in reversible reactions. Subsequently, we keep only reactions that are thermodynamically independent. Thermodynamic independence among reactions means that a reaction is only driven by its own thermodynamic force, which implies that the affinity of the reaction will be zero if and only if there is no change in its degree of advancement. This condition is usually fulfilled if we keep only elementary reactions (i.e., reactions that take place in one single step). As a consequence, we obtain a closed reaction set ℳ_0 _⊆ ℳ. Finally, we remove all species that are no longer involved with any of the reactions in ℳ_0_, leaving only the molecular species  associated with the closed subsystem.

The main rationale behind the second step is that the kinetic parameters **k **considered in this paper are assumed to be independent of the molecular concentrations. As a consequence, the values of these parameters will not change if the concentrations of the clamped species are allowed to vary. Therefore, we can construct a (possibly artificial) situation in which the concentrations of the clamped species vary as if they were dynamic species. Because our goal in creating the closed subsystem is to discover and enforce thermodynamic constraints on the kinetic parameters, we must include the clamped species in our model. This is contrary to simply removing all clamped species and the associated reactions, since this approach will not allow us to determine thermodynamically feasible values for the kinetic parameters of the removed reactions.

The third step is due to a simplification imposed on us by the current state of non-equilibrium thermodynamics. Thermodynamically dependent reactions influence each other, since the thermodynamic force of one reaction may drive the other reaction and vice versa. Unfortunately, it is not clear at this point how to deal with thermodynamically coupled reactions. Future research may be necessary to address this issue.

The resulting closed biochemical reaction subsystem is comprised of *N*_0 _molecular species  that interact through *M*_0 _coupled *reversible *reactions. The dynamic evolution of the molecular concentrations in this system is governed by:(2)

initialized by *x_n_*(0) = *q_n_*, for . We will characterize all reactions in ℳ_0 _by the generalized mass-action rate law [16]. In this case, the net fluxes are given by(3)

for *m *∈ ℳ_0_, where *r*_2*m*-1_, *r*_2*m *_are the (generalized) rate constants of the *m*^th ^forward and reverse reactions, respectively. The quantity *f_m_*[***x***(*t*), ***π***] can be any positive and finite function of the concentrations ***x***(*t*) and may depend on a set of kinetic parameters ***π***. For usual mass action kinetics, *f_m_*[***x***(*t*), ***π***] = 1. However, for more complex schemes, this function usually takes a rational or polynomial form. It is known that all reversible reaction rate laws in ideal mixtures (including reversible Michaelis-Menten kinetics) can be described by (3) [5]. Note that **k **includes the kinetic parameters ***π ***as well as the rate constants {*r*_2*m*-1_, *r*_2*m*_, *m *ℳ_0_}. It also includes the kinetic parameters of all reactions in ℳ_∈_ℳ_0_.

It is a direct consequence of thermodynamic analysis that a closed biochemical reaction system will asymptotically reach a *unique *non-zero state  of chemical equilibrium at which all concentrations become stationary (assuming, of course, we have non-zero initial conditions), satisfying the following detailed balance equations:(4)

As a consequence,(5)

These constraints must be satisfied by the rate constants in order for the closed biochemical reaction system to be thermodynamically feasible.

The constraints implied by (5) correspond to the reaction 'cycles' in the system. A reaction cycle is comprised of those reactions corresponding to the nonzero elements of a vector in the null space null () of the stoichiometry matrix  of the closed system. Clearly, (2) will be at a fixed point whenever the net fluxes of the underlying reactions are set equal to the corresponding elements of a vector in null (). If we denote by ***s ***the *N*_0 _*× *1 vector whose *n*^th ^element is the log steady-state concentration ln  and by ***z ***the *M*_0 _*× *1 vector whose *m*^th ^element is the log equilibrium constant *z_m _*= ln(*r*_2*m*-1_/*r*_2*m*_), then we can write (5) in a matrix-vector form as . Now, if ***b ***is a vector in the null space of , then  and , or(6)

which are the well-known Wegscheider conditions [6]. These conditions express necessary and sufficient constraints on the reaction rate constants of a closed biochemical reaction system to be thermodynamically feasible. Thus, if we denote the set of all thermodynamically feasible parameters **k **by , then any  satisfies (6) and, likewise, any **k **that satisfies (6) is a member of .

We want to emphasize that, in open biochemical reaction systems, the rate constants of the reversible reactions must also be constrained by the Wegscheider conditions, even if the system is far from equilibrium. To identify these constraints, we need to prune an open biochemical reaction system into a closed subsystem, by employing the technique discussed previously, and use the resulting stoichiometry matrix  to calculate the constraints given by (6).

An equally important observation is that the rate constants of the reactions pruned from an open system are not constrained by the Wegscheider conditions, since (4) must only be satisfied by the reactions in the closed subsystem. Furthermore, if a reaction *m *in a closed system is not part of a cycle, then *b_m _*= 0, for every ***b ***∈ null (), and its forward and reverse rate constants will not be thermodynamically constrained, since these constants trivially satisfy (6).

It can be shown (see Additional file 1) that the entropy production rate of an open biochemical reaction system at chemical equilibrium in which the net fluxes of the reactions in ℳ_0 _equal to the elements of a vector in the null space of the stoichiometry matrix , is given by(7)

where *A *= 6.02214084(18) × 10^23^mol^-1 ^is the Avogadro number, *V *is the system volume, and *k_B _*= 1.3806504 × 10^-23^JK^-1 ^is the Boltzmann constant. According to the second law of thermodynamics, the entropy production rate must always be greater than or equal to zero, with equality if and only if the system is at thermodynamic equilibrium. It is therefore clear from (7) that the Wegscheider conditions imply that the entropy production rate must be zero in this case (i.e., the system must be at thermodynamic equilibrium). As a consequence, the chemical motive force (which is the amount of energy added to the system per unit time due to mass exchange through its boundary) and the heat dissipation rate must also be zero. This makes intuitive sense, since a reaction cycle leaves all molecular concentrations unchanged and, therefore, there is no change in internal energy or mass flow through the system boundary. Clearly, we can think of the Wegscheider conditions as being a direct consequence of the thermodynamic requirement that *σ *(***b***) = 0, for every ***b ***∈ null ().

### Linear constraints

Unfortunately, (6) imposes a possibly infinite number of non-linear constraints on the rate constants of a closed biochemical reaction system. However, it is sufficient to satisfy (6) for *M*_2 _= *M*_0 _- *M*_1 _basis vectors {***b***^(*i*)^, *i *= 1, 2,..., *M*_2_} of the null space of , where *M*_0 _is the number of reactions and *M*_1 _= rank () (see Additional file 1). By using this observation and by taking logarithms on both sides of (6), we obtain the following *linear *constraints on the log-rate constants :(8)

where  is the *m*^th ^component of the *i*^th ^basis vector ***b***^(*i*)^. In Additional file 1 we derive an analytical formula for the basis vectors of the null space of  [see **Equation (S.1)**]. As a consequence, the possibly infinite non-linear Wegscheider conditions given by (6) are equivalent to much more manageable finite linear constraints on the log-values of the parameters of the closed subsystem, given by(9)

where ***κ ***:= ln(**k**) and  is an *M*_2 _× *J *matrix that can be easily constructed from knowledge of  using (8). Hence,  if and only if  ln(**k**) = 0.

We should note that there might be additional linear constraints that we may wish to impose on the logarithms of the kinetic parameters of a biochemical reaction system. Here are some examples:

• By using an appropriate experimental procedure, we may be able to accurately measure the equilibrium constant *R_m _*of the *m*^th ^reversible reaction. For a (generalized) mass-action reaction, we have *R_m _*:= *r*_2*m*-1_/*r*_2*m *_and thus we obtain a linear constraint  on the log rate constants of the *m*^th ^reaction, where  is the log value of the measured equilibrium constant.

• For a reversible Michaelis-Menten reaction, the Haldane relationship implies a linear constraint between the logarithms of the kinetic parameters of a reversible Michaelis-Menten reaction and [17].

• By using experimental techniques, such as plasmon resonance or atomic force microscopy, it may be possible to obtain a highly accurate measurement  of an individual kinetic parameter *k*_*j*_. In this case, we must impose the (trivial) constraint , where ***e***_*j *_is the *j*^th ^column of the *J *× *J *identity matrix.

• To reduce the dimensionality of parameter estimation, we may employ a sensitivity analysis approach, such as the one proposed in [18,19], to identify parameters that do not appreciably influence the cost of estimation. Determining accurate values for these parameters is inconsequential to the behavior of the biochemical reaction system at hand. Therefore, we can fix these parameters to some nominal values  throughout model calibration, resulting again in linear constraints of the form .

• We may want to expand an existing (validated) thermodynamically feasible model to include additional reactions and molecular species. We can do this by fixing the parameters of the existing model using linear constraints , where  is the *j*^th ^parameter value of the existing model. Then estimation takes place only on the parameters associated with the new reactions.

For a given biochemical reaction system, we can combine all possible linear constraints on the logarithms ***κ ***of the kinetic parameters **k **into a single matrix equation of the form:(10)

where  is an appropriately constructed *L *× *J *matrix and ***c ***is an *L *× 1 vector of known values determined by the constraints, with *L *being the number of constraints. Note that if there are no constraints other than the Wegscheider conditions, we would simply have  and ***c ***= **0**.

### Model calibration

We will now assume that we have obtained noisy measurements  of the concentration dynamics of selected molecular species  in an open biochemical reaction system of known stoichiometry, obtained by a set of distinct experiments  at discrete time points, within the observation time window . The problem of model calibration we consider in this paper is to determine *thermodynamically consistent *values for the kinetic parameters , so that the concentration dynamics  produced by the estimated system match ***y ***in some well-defined sense. Note that, for a given , the dynamics  are computed via (1) using initial conditions  that correspond to the *p*^th ^experimental condition. Data ***y ***may be obtained by appropriately designed *in vivo *or *in vitro *experiments, or by simulating an established and experimentally validated biochemical reaction model whose kinetic parameters are thermodynamically infeasible. Instead of focusing on the quality of the estimated values of the kinetic parameters, it has been argued in [20] that matching predicted and experimental observations of molecular concentrations is the right thing to do due to the 'sloppiness' of biochemical reaction systems (different combinations of parameter values may essentially lead to the same concentration dynamics).

There are many estimation techniques we can use to address the previous problem, such as maximum likelihood or Bayesian inference. The final product of these techniques is a cost function *C*(***κ ***|***y***) used to quantify the overall error between the predicted concentration measurements ***x***, obtained by simulating the biochemical reaction system with kinetic parameter values **k **= exp{***κ***}, and the noisy observations ***y***. In an effort to reduce the typically large dynamic range of kinetic parameter values, it is customary to estimate the log values ***κ ***instead of **k**. As a consequence, the problem of interest here is to compute an estimate  of the log kinetic parameters ***κ***, so that(11)

where  is the set of all ***κ***'s satisfying the linear constraints given by (10); i.e.,. For simplicity, we consider in this paper the least-squares error cost criterion, given by(12)

This error criterion is a consequence of a maximum likelihood approach to parameter estimation under the assumption of normally distributed observation errors. Note that the cost *C *depends on the log kinetic parameters ***κ ***through the molecular concentrations .

In this paper, we refer to the constrained optimization problem given by (11) as *Thermodynamically Consistent Model Calibration *(TCMC). The importance of TCMC lies on the formulation of the model calibration problem as one of *constrained *optimization via (11), with constraints that ensure at least the thermodynamic feasibility of the resulting model. A useful observation is that TCMC is agnostic to the choice of the cost function used and the algorithm employed for optimization. Moreover, we can easily transform the constrained optimization problem given by (11) to a standard unconstrained problem. Indeed, a well known result of linear algebra implies that , where , ***κ***_0 _is a *J × *1 vector that satisfies  is a *J *× *d *matrix whose columns form a basis for the null space of matrix , and ℜ*^d ^*is the space of all *d × *1 real valued vectors. Thus, if we can find a particular solution ***κ***_0 _to the constraints , we can reformulate the constrained optimization problem given by (11) as the following lower dimensional unconstrained problem:(13)

where(14)

Note that we assume here that there is more than one solution to . If only one solution exists, optimization is not necessary, since this solution will be our parameter estimate. On the other hand, if  has no solution, then we cannot find a ***κ ***that will simultaneously satisfy all necessary constraints, indicating that we must reformulate the problem.

The objective function *C*_0 _is non-convex with possibly many local minima. As a consequence, a gradient-based optimization algorithm for solving (13) may prematurely terminate at a local minimum with much larger cost than the globally minimum cost. To ameliorate this problem, we have decided in this paper to use a stochastic optimization algorithm, namely simulated annealing (SA) [21]. Stochastic optimization algorithms can move away from premature local minima, thus resulting in better solutions to optimization problems than when using deterministic techniques. Although many choices exist for optimization, such as the simultaneous perturbation stochastic approximation (SPSA) method employed in our previous work [11] and genetic algorithms, SA is a stochastic search optimization algorithm that enjoys several advantages over other algorithms. In particular, the most important features of SA are ease of implementation and the ability to avoid premature convergence by jumping away from local minima en route to finding a global minimum. In SA, a new value of ***v ***is proposed nearby the current value. The proposed value becomes the new value with a certain probability based on cost improvement. If the proposed value is not accepted, then the current value is used. The proposed value is usually drawn from an appropriately chosen probability distribution around the current value (e.g., a Gaussian distribution centered at the current value). See Additional file 1 for a detailed description of the SA algorithm used.

A natural question that arises here is whether different choices for ***κ***_0 _and  affect the final result of optimization. If we had an algorithm that could always find the global solution to a non-convex optimization problem, then the choice of ***κ***_0 _and  would have no effect on the solution. Since however global minima are difficult to find, we expect that different choices for ***κ***_0 _and  will have some impact on the final solution. Note that it would be advantageous to choose ***κ***_0 _as close as possible to the globally optimal solution. We attempt to do so in our subsequent example by taking ***κ***_0 _to be a solution to  that is closest (in the least-squares sense) to published values. On the other hand, we expect that the choice of  will have only a minor effect on optimization, since different matrices  amount to scaling or rotating the axes of the parameter space being searched. Good optimization algorithms, such as SA, are expected to be robust to such alterations.

### Example

We now demonstrate the proposed TCMC method by re-estimating the kinetic parameters of a classical model of the EGF/ERK signaling cascade [13]. This model consists of three compartments (extracellular space, cytoplasm, and endosomal volume), *N *= 100 biochemical species, and *M *= 125 reactions. Moreover, it is characterized by 90 different kinetic parameter values, a number that is smaller than the total number of individual reactions, due to the fact that some reactions share the same kinetic parameters, whereas, other reactions are not associated with any parameters.

Although the Schoeberl model has provided valuable insights into the biological mechanisms underlying EGF/ERK signaling, the values of the kinetic parameters published in the literature are thermodynamically infeasible. As a consequence, the concentration dynamics produced by the published model are physically impossible and could not occur in nature. By using TCMC to recompute thermodynamically feasible values for the kinetic parameters, we can construct a physically realizable model whose dynamics are expected to reflect the true behavior of EGF/ERK signaling more accurately than the dynamics produced by the published model.

We use the version of the Schoeberl model published in the BioModels database [22]. Moreover, we employ the same experimental time series data of ERK-PP activity used for creating the original model [13]. To simplify implementation of TCMC, we assume here that the Schoeberl model is characterized by *J *= 2*M *= 250 kinetic parameters (two parameters per reaction). For an irreversible reaction, we constrain the rate constant of the reverse reaction to be equal to zero. For those reactions not associated with any kinetic parameters, we assign two artificial parameters (one for the forward and one for the reverse reaction) and constrain both their values to be zero.

We implement the following TCMC procedure to re-estimate the values of the kinetic parameters in a thermodynamically consistent manner. First, we find the closed subsystem of the Schoeberl model (see Additional file 1). This subsystem consists of *N*_0 _= 93 molecular species and *M*_0 _= 83 reversible elementary (monomolecular and bimolecular) reactions. Then, we determine the thermodynamic constraints by using the 93 *× *83 stoichiometry matrix  of the closed subsystem. It turns out that the rank of the stoichiometry matrix  is 65. As a consequence, the closed subsystem contains *M*_2 _= 83 - 65 = 18 independent reaction cycles, determined by the columns of matrix , given by **Equation (S.1) **of Additional file 1 (see the file for details on the reaction cycles). Therefore, the rate constants of the closed subsystem must satisfy 18 independent Wegscheider conditions. It turns out that the published Schoeberl model satisfies only 10 of these conditions. As a matter of fact, the entropy reaction rates, given by (7), associated with 5 independent reaction cycles are negative, in direct violation of the second law of thermodynamics, whereas, the entropy reaction rates of 3 reaction cycles are positive, with the remaining being equal to zero (see **Table S.2 **in Additional file 1).

Next, we construct matrix  and vector ***c ***by combining the 18 thermodynamic constraints with 167 linear equality constraints originally built into the model that relate various parameters across reactions (see Additional file 1 for details on these constraints), thus producing the linear constraints . In this case,  is a 185 *× *250 matrix, whereas, ***c ***is a 250 *× *1 vector with elements 0 or -*∞ *(this corresponds to kinetic parameters whose values are fixed to zero). It turns out that rank () = 171, which implies that the dimension of the null space of  is *d *= 79. Subsequently, we find a basis for the null space of  and form the 250 *× *79 matrix . Moreover, we find a particular solution ***κ***_0 _of  that is closest, in the least-squares sense, among all other solutions to the published thermodynamically infeasible parameter values. We accomplish this by using a well-known approach for solving constrained least-squares problems [23]. Finally, by using (12) on the aforementioned experimental time series data and SA, we calculate the 79 *× *1 vector  by minimizing the cost function *C*_0_(***v ***|***y***), given by (14), and set .

We take the thermodynamically feasible log kinetic parameter values ***κ***_0 _to be close to the published kinetic parameter values, since the published values already produce a good match between the experimentally available and predicted molecular dynamics. In this case, TCMC provides a thermodynamically consistent correction of ***κ***_0_, by means of , that reduces the cost of estimation, thus further improving the match between the experimentally obtained and predicted dynamics.

**Figure 1:**
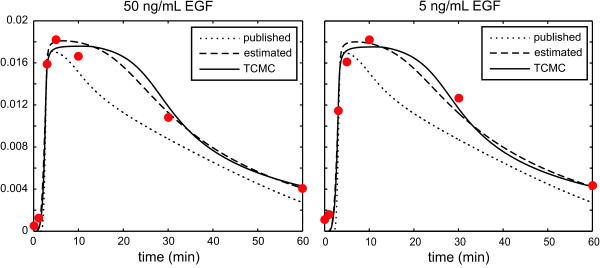
**ERK-PP concentration dynamics, measured in mol/m^3^, under two different input EGF concentrations**. The Additional file 1 reports additional dynamics.

In Figure 1, we depict the concentration dynamics of ERK-PP obtained by the published model (dotted curves), for two different input EGF concentrations, namely 50 ng/mL and 5 ng/mL, whereas, in **Figure S1 **of Additional file 1 we depict the concentration dynamics of ERK-PP obtained by the published model for three additional concentrations of input EGF, namely 0.5 ng/mL, 0.125 ng/mL, and 0.0625 ng/mL. Clearly, the resulting dynamics match the available densitometric data (indicated by the circles) rather poorly.

We should note here that the ERK-PP dynamics originally published in [13] seem to match the available data pretty well - see Figure 2F in [13]. However, the model detailed in the original publication cannot be used to reproduce the results. On the other hand, the model available in the BioModels database does not reproduce the results published in the Schoeberl paper but produces dynamics that are very similar to the ones reported in that paper.

The solid curves in Figure 1 and **Figure S1 **of Additional file 1 depict the ERK-PP concentration dynamics estimated by TCMC. TCMC results in a thermodynamically consistent model of the EGF/ERK signalling cascade that produces ERK-PP concentration dynamics which match the available experimental data noticeably better than the dynamics produced by the published model. As a matter of fact, model fit between predicted and experimentally measured ERK-PP concentration dynamics, quantified by the least-squares error given by (12), is reduced by 69%. TCMC simultaneously adjusts the values of the kinetic parameters in order to minimize the cost of fitting the ERK-PP response to the available densitometric data. This 'collective fitting' strategy has been recognized in the literature [20] as being more desirable than constructing a biochemical reaction system model from individual parameter estimates in a piecewise fashion, which is the case with the published Schoeberl model.

To separate the effect of collective fitting versus imposing the underlying thermodynamic constraints on the kinetic parameters, we use the same simulated annealing algorithm employed by TCMC to estimate the kinetic parameters without including the thermodynamic constraints. The resulting (thermodynamically infeasible) estimated dynamics are depicted by the dashed curves in Figure 1 and **Figure S1 **of Additional file 1. As expected, these dynamics fit the densitometric data better than the dynamics obtained by the published model. As a matter of fact, model fit between predicted and experimentally measured ERK-PP concentration dynamics is reduced by 70% in this case, which is slightly better than the one obtained by TCMC. It will shortly become clear however that the solution obtained without imposing the thermodynamic constraints leads to unrealistic system behavior. In the following, we discuss a number of advantages gained by TCMC over estimating the kinetic parameters using a collective fitting approach that does not consider the underlying thermodynamic constraints.

## Discussion

### Qualitative/quantitative value of thermodynamic consistency

Due to lack of thermodynamic consistency in the parameter values of the published Schoeberl model, the molecular dynamics produced by this model cannot possibly occur in nature. Because the values estimated by the proposed TCMC method satisfy all necessary thermodynamic constraints, it is expected that the resulting TCMC model will provide a more accurate representation of EGF/ERK signaling than the published Schoeberl model.

To provide an example of a potentially important difference between the published model and the TCMC model calculated in this paper, we consider the *integrated response *of ERK-PP activity (i.e., the cumulative ERK-PP concentration). It has been argued in the literature that the integrated response provides an appropriate metric for quantifying dependence of DNA synthesis on ERK activation in certain cells [24]. As a consequence, this feature of the ERK-PP concentration dynamics can influence a number of diverse biologically outcomes, such as cell cycle progression, cell proliferation, and cell differentiation.

**Figure 2:**
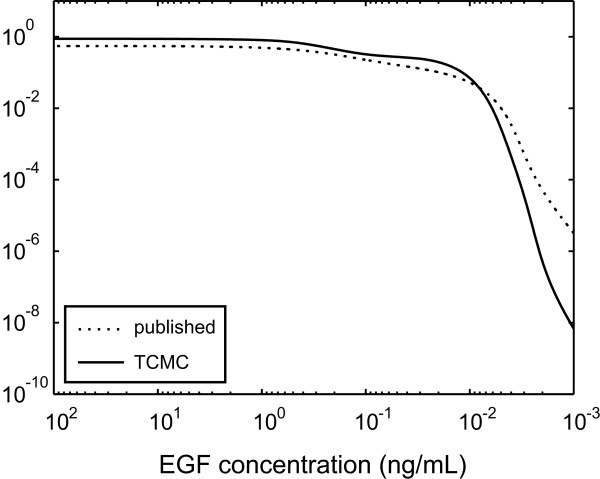
**Integrated response of ERK-PP activity, measured in mol · min/m^3^, as a function of input EGF concentrations**.

In view of the fact that differences in the integrated response of ERK-PP activity may cause distinct biological outcomes, it is reasonable to believe that a key objective of EGF/ERK signaling is to maintain robust integrated response to changes in input EGF concentration while producing a quick and sharp 'switch-like' transition between states of differing biological outcomes. In Figure 2, we provide a log-log plot of the integrated response of ERK-PP activity predicted by the published (dotted curve) and TCMC (solid curve) models, for a wide range of input EGF concentrations. Although the integrated response predicted by both models is indeed robust for input EGF concentrations larger than 10^-2 ^ng/mL, when the EGF concentration decreases below 10^-2 ^ng/mL, the integrated response of ERK-PP activity predicted by the TCMC model exhibits a sharper transition from large to small values than the one predicted by the published model. As a matter of fact, the TCMC model predicts seven orders of magnitude decrease in the integrated response, when the input EGF concentration decreases from 10^-2 ^ng/mL to 10^-3 ^ng/mL, whereas, the published model predicts only four orders of magnitude decrease. By considering the discussion in [24], we believe that this behavior by the TCMC model may turn out to be a biologically important property of the EGF/ERK signaling pathway that cannot be effectively captured by the published model.

**Figure 3:**
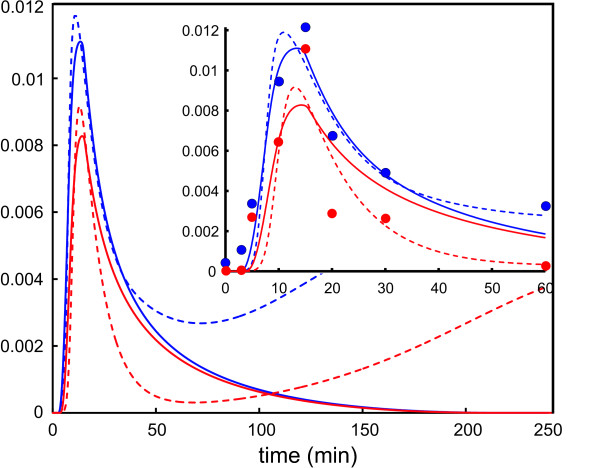
**Long-term ERK-PP concentration dynamics, measured in mol/m^3^, predicted by the estimated thermodynamically infeasible model (dashed curves) and the TCMC model (solid curves) under 0.125 ng/mL (blue curves) and 0.0625 ng/mL (red curves) input EGF concentrations**. The inset shows the short-term behavior predicted by the two models as well as the corresponding densitometric data (blue and red circles).

We will now show that the TCMC model may result in a biologically plausible prediction of ERK-PP activity that can also be different than the one produced by the estimated thermodynamically infeasible model using collective fitting. In Figure 3, we show the long-term behavior of the estimated thermodynamically infeasible model. Under certain normal biological conditions (e.g., typical EGF receptor concentration, such as the one considered in our paper), ERK-PP activity is expected to decay to zero at steady-state [25]. However, one can see from Figure 3 that, even after 4 hours, the concentration of ERK-PP predicted by the estimated thermodynamically infeasible model is steadily increasing - possibly being driven by the chemical perpetuum mobiles that occur when the Wegscheider conditions are violated. This unrealistic behavior appears despite the fact that the transient dynamics, depicted in the inset of Figure 3 by the dashed curves, fit the data better than the TCMC dynamics, denoted by the solid curves. Such a sustained and long lasting response may lead to different biological outcomes than the ones resulting from ERK-PP activity that decays to zero at steady-state [25]. Thus, the estimated thermodynamically infeasible model can lead to erroneous biological predictions, despite its reasonable fit to the available densitometric data.

Our previous examples show that thermodynamic consistency may result in model behavior that is different than the one predicted by thermodynamically infeasible models of cellular function. However, more research is needed to experimentally validate observed differences and demonstrate that lack of thermodynamic consistency may indeed result in inaccurate (or even false) biological predictions.

### Flux analysis

Flux-based analysis of biochemical reaction systems is a widely used method for understanding the principles underlying the production and regulation of mass flow in cellular systems, such as signaling or metabolic pathways. It turns-out that the Wegscheider conditions, given by (6), constrain the reaction fluxes. If flux analysis does not take into account these constraints, then it may lead to inaccurate or misleading conclusions about the behavior and properties of mass flow in biochemical reaction systems.

If {***b***^(*i*)^, *i *= 1, 2,..., *M*_2_} is a set of basis vectors of the null space of the stoichiometry matrix  of the closed subsystem, and  are respectively the forward and reverse fluxes of the *m*^th ^reaction at time *t*, then (see Additional file 1 and [6])(15)

where  is the *m*^th ^element of the basis vector ***b***^(*i*)^. Note that this equation must be satisfied at each time point , even far away from equilibrium. Moreover, it is satisfied for any vector ***b ***in the null space of .

Since TCMC always leads to a thermodynamically feasible biochemical reaction system with parameters satisfying the Wegscheider conditions, the flux constraints imposed by (15) are satisfied as well. Thus, thermodynamically consistent flux analysis can be performed on the resulting system without any additional considerations, and the behavior of the system is always physically realizable.

### Bias-variance tradeoff and overfitting

In addition to the previous advantages, there is an important statistical benefit for thermodynamically constraining the parameters of a biochemical reaction system. By searching for kinetic parameter values within a thermodynamically consistent subset of the parameter space, we may reduce the variance of estimation and thus lower the estimation error through the well-known bias-variance tradeoff.

The mean-square error (MSE)  in cost, where  is an estimator of the 'true' parameters ***κ***_true_, satisfies:

Generally speaking, imposing constraints on the parameters may increase the bias term but decrease the variance. However, since the true parameter values must satisfy the thermodynamic constraints, we expect a decrease in variance without an increase in bias. As a consequence, searching for parameter values within the thermodynamically consistent subspace of the parameter space may lead to a lower mean square error in cost due to smaller variance. Since the volume of a search space grows exponentially in the dimension of the space, gains in variance (and hence improvements in the mean square error) are expected to be large.

A related statistical notion in estimation problems is data *overfitting*. Overfitting refers to situations where model complexity (i.e., number of parameters) is high and the amount and quality of available data is comparatively low. In this case, it is often possible that we match the data so well that, in addition to matching the underlying physical phenomena of interest, the model fits the measurement noise as well. This situation reduces the predictive power of the estimated model. The relationship of overfitting to the bias-variance tradeoff is clear: complex models are characterized by low bias and are able to describe a wide range of phenomena but suffer from high variance in parameter estimation (i.e., different data sets may lead to wildly different parameter estimates via overfitting).

Most often, the behavior of biochemical reaction systems is only influenced by a small number of parameters (due to robustness of such systems to the underlying kinetic parameters). This reduces the actual number of parameters that must be estimated with precision. Moreover, the thermodynamic constraints further reduce the number of parameters to be estimated, alleviating some overfitting concerns. Imposing additional parameter constraints, such as the ones employed by the Schoeberl model, may further be used to combat this issue. Unfortunately, model complexity is much higher than the amount and quality of available data in most problems of systems biology and overfitting remains a major concern even when using TCMC. In the example considered in this paper, time series data is only available for one crucial chemical species. As a consequence, it is natural to expect that the dynamics produced by the TCMC model overfit the available data to a certain extent. Thus, when more experimental data become available, TCMC must be rerun in order to produce a better calibration of the model, with a new cost function that includes the additional data.

In light of these concerns, some may argue that collective fitting of model parameters is not the correct approach, and that a reductionist approach is more appropriate (i.e., attempting to measure parameters individually and then combine the results to determine an appropriate model calibration). Unfortunately, the reductionist approach is time consuming, extremely expensive, and, in most cases, impossible with current experimental techniques. Moreover, incorrect implementation of a reductionist approach may lead to a thermodynamically infeasible model calibration. This is clearly the case with the Schoeberl model (and most probably with other models published in the literature).

TCMC is a collective fitting procedure, but offers a pragmatic compromise to the reductionist approach. In light of the fact that some parameters may be measured individually with extreme precision, TCMC allows for these parameters to be fixed to their measured values using matrix . In addition, a more advanced Bayesian cost function can be used (e.g., see [11]) to factor in prior experimental knowledge when parameters have been previously estimated with less precise experimental techniques.

### Computational advantages

According to the 'curse of dimensionality,' which refers to an exponential increase in the volume of the parameter space as its dimension grows, estimation becomes substantially harder in high dimensional spaces. A 'naïve' search of that space, in an effort to find the 'true' parameter values of a biochemical reaction system [assuming that these values minimize the cost function given by (12)], is hopeless. As a matter of fact, the probability of obtaining parameter values that satisfy the Wegscheider conditions and other underlying log-linear constraints by uniformly sampling the entire parameter space (which is an 'easier' problem than finding the 'true' parameter values) is zero. As a consequence, the constraints must be explicitly considered by the optimization problem at hand to have any hope of successfully solving the problem of model calibration.

As a matter of fact, since the feasible manifold is of lower dimension than the entire parameter space, methods that do not consider the underlying thermodynamic and non-thermodynamic constraints will spend most time searching the immense infeasible portions of the parameter space. The imposition of constraints among the kinetic parameters of a biochemical reaction system reduces the dimensionality of the parameter space to a smaller feasible region and make parameter estimation computationally easier. TCMC makes this explicit, by performing optimization over a lower dimensional space, spanned by the lower dimensional vectors ***v***, instead of the entire parameter space, spanned by the higher dimensional vectors ***κ***.

## Conclusions

For a biochemical reaction system to be physically realizable, it is required that the underlying kinetic parameters satisfy certain thermodynamic constraints, known as Wegscheider conditions. This issue has been largely ignored in the literature, as evidenced by the fact that many published models violate these constraints. The model calibration method we have proposed in this paper can be effectively used to determine thermodynamically consistent values for the kinetic parameters of any set of reactions in an ideal homogeneous mixture at constant temperature and volume. Our method is simple to understand and implement. Moreover, it can be easily incorporated into any existing or newly proposed calibration technique in order to make sure that the resulting model satisfies the fundamental laws of thermodynamics as well as other desirable conditions and constraints.

There are two major issues associated with calibrating biochemical reaction systems:

1. The quality and quantity of available data are inadequate to allow sufficient estimation of all underlying parameter values.

2. Biochemical reaction models contain many parameters whose numbers dramatically increase with model size and detail. As a consequence, the curse of dimensionality seriously hampers estimation algorithms.

The first issue is primarily associated with current limitations of experimental methods and approaches. To address this issue, we need substantial improvements in experimental equipment and methodologies. However, TCMC scales well with future improvements in data quality and quantity. Matrix  can handle arbitrary parameter measurements (equilibrium constants, Haldane relations, direct kinetic parameter measurements, etc.). Moreover, TCMC can employ any cost function of choice, so additional concentration data can be incorporated seamlessly therein.

The second issue is the largest obstacle facing model calibration techniques. To reduce dimensionality, we must attempt to exploit mathematical structure particular to the biological problem at hand. TCMC attempts to address this problem in two ways:

• First, TCMC uses the fact that there are fundamental physical principles underlying biochemical reaction systems that may constrain the set of possible kinetic parameter values. As a consequence of the fundamental laws of thermodynamics, most complex biochemical networks contain reaction cycles that constrain the kinetic parameters according to (9). These constraints allow TCMC to reduce dimensionality by restricting the estimation problem on a smaller thermodynamically feasible subset of the parameter space.

• Second, experimental data and mathematical analysis can often provide other forms of constraints on the underlying parameters (e.g., through directly measuring rate or equilibrium constants, by determining Haldane relationships between enzymatic parameters, etc.). In particular, sensitivity analysis may reveal non-influential kinetic parameters that can be set to some nominal values without appreciably affecting system behavior. All these additional constraints can be accounted for by the  matrix in (10), further reducing the dimensionality of the ensuing parameter estimation problem.

As we mentioned before, dimensionality reduction is made explicit by TCMC, since optimization takes place over a smaller dimensional vector ***v***, instead of the higher dimensional vector ***κ ***specified by the model. As a consequence, TCMC does not entirely rely on finding the globally optimal parameter values that best fit available dynamic data of molecular concentration. Imposing thermodynamic (and other log-linear) constraints allows TCMC to restrict its search for appropriate parameter values over a smaller subspace of the entire parameter space in order to reach a compromise between optimal data fit and biophysical feasibility. It has been recently pointed out in the literature that this approach to parameter estimation should be considered as an important part of determining the parameter values of complex biological models [26].

Recently, a method has been proposed in the literature for inferring a complete and consistent set of kinetic parameter values from incomplete and inconsistent data [27]. This method, known as 'parameter balancing,' employs a Bayesian estimation approach based entirely on published data pertaining the values of the underlying kinetic parameters. Although parameter balancing can be used to provide thermodynamically consistent values for the kinetic parameters of a biochemical reaction system, the method does not include quantitative dynamic measurements of molecular concentrations. As a consequence, parameter balancing may result in a thermodynamically feasible biochemical reaction model that does not adequately predict experimental observations of dynamic system behavior. Future research may focus on combining TCMC with parameter balancing to utilize published parameter sets as well as dynamic experimental data.

A problem that we have not addressed in this paper is the influence of ions, such as K^+^, and Ca^2+^, and certain environmental factors, such as the temperature and pH, on the thermodynamic behavior of a biochemical reaction system [28]. Our objective in this paper is not to address biochemical reaction systems with this level of complexity, but to focus on the widely reported simpler models of cellular function that consider only interactions among biochemical reactants in a fixed environment. Note, however, that temperature and pH dependence of parameters has been accounted for in [27] via parameter balancing using log-linear equations between biochemical parameters. Since arbitrary log-linear constraints between parameters can be enforced by TCMC via (10), we suspect that TCMC can be used directly or appropriately modified to handle additional biochemical complexities that have not been addressed in this paper.

Another problem that we have not addressed here is constructing new biochemical reaction models of cellular function. Since, in this paper, we only address the model calibration problem, we take (1) as given and proceed to determine the parameter vector **k **from data. In general, determining the structure (i.e., the stoichiometry) of a biochemical reaction network is an extremely laborious task. Preliminary work indicates that thermodynamics can also play a key role in estimating the structural complexity of biochemical reaction systems [29]. Future scientific investigations are necessary to further examine this open problem.

## Authors' contributions

GJ developed the general methodology and coded a substantial portion of the software. JG derived many theoretical results and ideas and wrote substantial portions of the final document. GJ and JG both interpreted the obtained computational results and approved the final version of the paper.

## Supplementary Material

Additional file 1 **In this document, we provide supplementary mathematical and computational details required to fully understand the material presented in the Main Text**. Click here for file
