# Supplementary material for: Thermodynamically consistent model calibration in chemical kinetics
Source: BMC Syst Biol. 2011 May 6;5:64. doi: 10.1186/1752-0509-5-64 (PMC3117730; doi:10.1186/1752-0509-5-64)
Supplement: Additional file 1 — In this document, we provide supplementary mathematical and computational details required to fully understand the material presented in the Main Text. [file 1752-0509-5-64-S1.PDF]

SUPPLEMENTARY MATERIAL

Thermodynamically Consistent Model Calibration in Chemical Kinetics

Garrett Jenkinson, John Goutsias*

Whitaker Biomedical Engineering Institute, The Johns Hopkins University, Baltimore, MD 21218

Email: Garrett Jenkinson - jenkinson@jhu.edu; John Goutsias*- goutsias@jhu.edu;

*Corresponding author

Thermodynamic Constraints

Finite set of thermodynamic conditions

We show that, if the Wegscheider conditions are satisfied for $M_2 = M_0 - M_1$ basis vectors $\{\boldsymbol{b}^{(i)}, i = 1, 2, \ldots, M_2\}$ of the null space of $\mathbb{S}_0$, where M_0 is the total number of (reversible) reactions in a closed biochemical reaction system with stoichiometry matrix $\mathbb{S}_0$ and $M_1 = \text{rank}(\mathbb{S}_0)$, then they will also be satisfied for all $\boldsymbol{b} \in \text{null}(\mathbb{S}_0)$.

Note that, for any $\boldsymbol{b} \in \text{null}(\mathbb{S}_0)$, we have that

$$\boldsymbol{b} = \sum_{i=1}^{M_2} a_i \, \boldsymbol{b}^{(i)},$$

for some scalar coefficients $a_i, i = 1, 2, \ldots, M_2$. As a consequence, and from Equations (5) and (6) in the Main Text, we have that

$$\begin{aligned}
\ln \prod_{m \in \mathcal{M}_0} \left(\frac{r_{2m-1}}{r_{2m}} \right)^{b_m} &= \sum_{m \in \mathcal{M}_0} b_m \ln \frac{r_{2m-1}}{r_{2m}} \\
&= \sum_{m \in \mathcal{M}_0} b_m z_m \\
&= \sum_{m \in \mathcal{M}_0} \left[\sum_{i=1}^{M_2} a_i b_m^{(i)} \right] z_m
\end{aligned}$$

$$
\begin{aligned}
&= \sum_{i=1}^{M_2} a_i \sum_{m \in \mathcal{M}_0} b_m^{(i)} z_m \\
&= \sum_{i=1}^{M_2} a_i \sum_{m \in \mathcal{M}_0} b_m^{(i)} \ln \frac{r_{2m-1}}{r_{2m}} \\
&= \sum_{i=1}^{M_2} a_i \ln \prod_{m \in \mathcal{M}_0} \left(\frac{r_{2m-1}}{r_{2m}} \right)^{b_m^{(i)}} \\
&= 0,
\end{aligned}
$$

for every $\boldsymbol{b} = \{b_m, m \in \mathcal{M}_0\} \in \text{null}(\mathbb{S}_0)$, since the Wegscheider conditions are assumed to be satisfied by the basis vectors $\{\boldsymbol{b}^{(i)}, i = 1, 2, \ldots, M_2\}$ of the null space of $\mathbb{S}_0$. This shows that the Wegscheider conditions are also satisfied for every $\boldsymbol{b} \in \text{null}(\mathbb{S}_0)$.

Analytic form of stoichiometric basis

We can rearrange the columns and rows of the stoichiometry matrix $\mathbb{S}_0$ (by appropriately relabeling the molecular species and reactions) so that the first M_1 columns are linearly independent, whereas, the remaining M_2 columns linearly dependent on the first columns. In this case, we can write the $N_0 \times M_0$ stoichiometry matrix $\mathbb{S}_0$ in the following block matrix form:

$$
\mathbb{S}_0 = \begin{bmatrix} \mathbb{S}_{11} & \mathbb{S}_{12} \\ \mathbb{S}_{21} & \mathbb{S}_{22} \end{bmatrix},
$$

where $\mathbb{S}_{11}$ is an *invertible* $M_1 \times M_1$ matrix, whereas, $\mathbb{S}_{12}$, $\mathbb{S}_{21}$, and $\mathbb{S}_{22}$ are $M_1 \times M_2$, $(N_0 - M_1) \times M_1$, and $(N_0 - M_1) \times M_2$ matrices, respectively. It is a well-known fact [1] that the general solution of $\mathbb{S}_0 \boldsymbol{b} = \boldsymbol{0}$ is given by $\boldsymbol{b}' = -\mathbb{S}_{11}^{-1} \mathbb{S}_{12} \boldsymbol{b}''$, for an arbitrary $\boldsymbol{b}''$, where $\boldsymbol{b}'$, $\boldsymbol{b}''$ are $M_1 \times 1$ and $M_2 \times 1$ vectors, respectively, such that

$$
\boldsymbol{b} = \begin{bmatrix} \boldsymbol{b}' \\ \boldsymbol{b}'' \end{bmatrix}.
$$

This implies that the columns of matrix

$$
\mathbb{B}_0 := \begin{bmatrix} -\mathbb{S}_{11}^{-1} \mathbb{S}_{12} \\ \mathbb{I} \end{bmatrix}, \tag{S.1}
$$

where $\mathbb{I}$ is the $M_2 \times M_2$ identity matrix, form a basis for the null space of $\mathbb{S}_0$. As a consequence of this result and the fact that the Wegscheider conditions are satisfied so long as they are satisfied by a set of basis

vectors of null($\mathbb{S}_0$), we can conclude that the Wegscheider conditions, given by Equation (6) in the Main Text, are equivalent to the following conditions [2]:

$$\varrho_{2m'} - \varrho_{2m'-1} + \sum_{m \in \mathcal{M}_1} [\mathbb{S}_{11}^{-1}\mathbb{S}_{12}]_{m,m'} (\varrho_{2m-1} - \varrho_{2m}) = 0, \quad \text{for every } m' \in \mathcal{M}_2, \tag{S.2}$$

where $\mathcal{M}_1 = \{1, 2, \ldots, M_1\}$, $\mathcal{M}_2 = \{M_1 + 1, M_1 + 2, \ldots, M_0\}$, $\varrho_{2m-1} = \ln r_{2m-1}$, $\varrho_{2m} = \ln r_{2m}$, and $[\mathbb{S}_{11}^{-1}\mathbb{S}_{12}]_{m,m'}$ is the element of the mth row and the m'^{th} column of matrix $\mathbb{S}_{11}^{-1}\mathbb{S}_{12}$. Equation (S.2) implies that the log-rate constants of a closed biochemical reaction system must satisfy the linear constraints given by Equation (9) in the Main Text, where $\mathbb{W}$ is an appropriately constructed [by means of (S.2)] $M_2 \times J$ matrix.

Flux constraints and entropy production

If $\boldsymbol{b}$ is a vector in the null space of the stoichiometry matrix $\mathbb{S}_0$ of the closed subsystem of an open biochemical reaction system, and $\phi_m^+(t, \mathbf{k})$, $\phi_m^-(t, \mathbf{k})$ are respectively the forward and reverse fluxes of the mth reaction at time t, given by

$$\phi_m^+(t, \mathbf{k}) = f_m[\boldsymbol{x}(t), \boldsymbol{\pi}]\, r_{2m-1} \prod_{n \in \mathcal{N}_0} [x_n(t)]^{\nu_{nm}} \quad \text{and} \quad \phi_m^-(t, \mathbf{k}) = f[\boldsymbol{x}(t), \boldsymbol{\pi}]\, r_{2m} \prod_{n \in \mathcal{N}_0} [x_n(t)]^{\nu'_{nm}},$$

then

$$\begin{aligned}
\sum_{m \in \mathcal{M}_0} b_m \ln \frac{\phi_m^+(t, \mathbf{k})}{\phi_m^-(t, \mathbf{k})} &= \sum_{m \in \mathcal{M}_0} b_m \ln \left[\frac{r_{2m-1}}{r_{2m}} \prod_{n \in \mathcal{N}_0} [x_n(t)]^{-s_{nm}} \right] \\
&= \sum_{m \in \mathcal{M}_0} b_m \ln \frac{r_{2m-1}}{r_{2m}} + \ln \prod_{n \in \mathcal{N}_0} [x_n(t)]^{-\sum_{m \in \mathcal{M}_0} s_{nm} b_m} \\
&= \ln \prod_{m \in \mathcal{M}_0} \left(\frac{r_{2m-1}}{r_{2m}} \right)^{b_m} \\
&= 0, \quad \text{for } i = 1, 2, \ldots, M_2, \quad t \in \mathcal{T},
\end{aligned} \tag{S.3}$$

where b_m is the mth element of $\boldsymbol{b}$. Equation (S.3) is due to the Wegscheider conditions, given by Equation (6) in the Main Text, and the fact that $\mathbb{S}_0 \boldsymbol{b} = \mathbf{0}$. This result shows that the fluxes of a biochemical reaction system must be constrained by Equation (15) in the Main Text.

It can be shown that a biochemical reaction system is governed by the following balance equations [3]:

$$\frac{dS(t)}{dt} = \sigma(t) - \frac{h(t)}{T} \quad \text{and} \quad \frac{dG(t)}{dt} = f(t) - T\sigma(t), \tag{S.4}$$

where $S(t)$ is the entropy of the system at time t, $G(t)$ is the free energy stored in the system at time t, $\sigma(t)$ is the entropy production rate, $h(t)$ is the heat dissipation rate, $f(t)$ is the chemical motive force, and T is the temperature. The entropy production rate is given by

$$\sigma(t) \;=\; AVk_B \sum_{m \in \mathcal{M}_{\text{in}} \cup \mathcal{M}_{\text{ex}}^{(r)}} [\phi_m^+(t,\mathbf{k}) - \phi_m^-(t,\mathbf{k})] \ln \frac{\phi_m^+(t,\mathbf{k})}{\phi_m^-(t,\mathbf{k})} \,, \qquad \text{(S.5)}$$

where A is the Avogadro number, V is the system volume, k_B is the Boltzmann constant, $\mathcal{M}_{\text{in}}$ is the set of all *internal* (and necessarily reversible) reactions, $\mathcal{M}_{\text{ex}}^{(r)}$ is the set of all *exchange* reversible reactions, and $\phi_m^+(t,\mathbf{k})$, $\phi_m^-(t,\mathbf{k})$ are the forward and reverse fluxes of the mth reaction. A reaction is internal if it involves only dynamic species as reactants and products, or dynamic and clamped species but for which a clamped species is both reactant and product of the reaction with equal stoichiometry (i.e., it is a catalyst). A reaction is an exchange reaction if its occurrence involves the consumption or production of a clamped molecular species. Note that occurrence of such a reaction requires that an equal number of molecules of the clamped species are transferred in or out of the system through its boundary in order to make sure that their concentrations remain constant.

It is not difficult to see that the set $\mathcal{M}_0$ of all reactions in the closed reaction subsystem, obtained by the technique discussed in the Main Text, equals to $\mathcal{M}_{\text{in}} \cup \mathcal{M}_{\text{ex}}^{(r)}$. As a consequence, (S.3) and (S.5) imply that the entropy production rate of an open biochemical reaction system at chemical equilibrium in which the net fluxes of all reactions in $\mathcal{M}_0$ equal to b_m, for $m \in \mathcal{M}_0$, where $\boldsymbol{b} \in \text{null}(\mathbb{S}_0)$, is given by

$$\sigma(\boldsymbol{b}) \;=\; AVk_B \, \ln \prod_{m \in \mathcal{M}_0} \left(\frac{r_{2m-1}}{r_{2m}} \right)^{b_m}, \qquad \text{for all } \boldsymbol{b} \in \text{null}(\mathbb{S}_0),$$

which shows Equation (7) in the Main Text.

The second law of thermodynamics postulates that an increase in the entropy of a biochemical reaction system must always be larger than the heat absorbed by the system divided by the temperature. This implies that

$$\frac{dS(t)}{dt} \geq -\frac{h(t)}{T}, \qquad \text{for all } t \geq 0.$$

This inequality, together with (S.4), implies that

$$\sigma(t) \geq 0, \qquad \text{for all } t \geq 0, \qquad \text{(S.6)}$$

which is already guaranteed by (S.5), as well as

$$\frac{dG(t)}{dt} \leq f(t), \qquad \text{for all } \ t \geq 0.$$

At chemical equilibrium, (S.4) and (S.6) imply that

$$0 \leq T\overline{\sigma} = \overline{h} = \overline{f},$$

where $\overline{\sigma}$, $\overline{h}$, and $\overline{f}$ are the steady-state entropy production rate, heat dissipation rate, and chemical motive force, respectively. Equality holds if and only if the system is at thermodynamic equilibrium (i.e., at a state of chemical equilibrium in which the steady-state entropy production rate, heat dissipation rate, and chemical motive force are all equal to zero). Clearly, the Wegscheider conditions, given by Equation (6) in the Main Text, imply that the entropy production rate $\sigma(\boldsymbol{b})$ must be zero (i.e., the system must be at thermodynamic equilibrium). As a consequence, the chemical motive force $f(\boldsymbol{b})$ and the heat dissipation rate $h(\boldsymbol{b})$ must be zero as well.

EGF/ERK Signaling Cascade

The EGF/ERK signaling cascade model we use in this paper has been suggested by Schoeberl *et. al.* [4] and can be found in the publicly available BioModels database [5]. This model consists of three compartments (extracellular space, cytoplasm, and endosomal volume), 100 molecular species, and 125 biochemical reactions.

The proposed TCMC method requires that we manually find the closed subsystem of a biochemical reaction system by following the rules discussed in the Main Text. To determine the closed subsystem associated with the EGF/ERK signaling cascade model, we first need to remove all 42 reactions summarized in Table S1 for the stated reasons. Then, we must allow the concentration of the only clamped molecular species in the system, namely EGF, to fluctuate freely as a function of time. In this and subsequent tables, we employ the labeling for the reactions and the associated kinetic parameters used in the original publication [4]. The remaining 83 reactions compose the closed reaction set $\mathcal{M}_0$. Moreover, the 93 molecular species associated with the reactions in $\mathcal{M}_0$ make up the set $\mathcal{N}_0$. Now, we can construct the 93×83 stoichiometry matrix $\mathbb{S}_0$ of the closed subsystem by including only the reactions in $\mathcal{M}_0$ and the species in $\mathcal{N}_0$. It turns out that the dimension of null$(\mathbb{S}_0)$ is $d = 18$. This implies that the closed EGF/ERK subsystem contains 18 independent reaction cycles, associated with the columns of matrix $\mathbb{B}_0$ given

by (S.1), and that the rate constants are constrained by 18 independent Wegscheider conditions. We depict the reactions associated with each independent cycle in Table S.2. In this table, we also depict the entropy production rates of the independent reaction cycles associated with the published Schoeberl model, given by Equation (7) in the Main Text.

An attractive feature of TCMC is its ability to incorporate linear non-thermodynamic constraints into estimation alongside the thermodynamic constraints imposed by the Wegscheider conditions. In Table S3, we mark with boldface the kinetic parameters whose values have been constrained in the original Schoeberl model. These 167 equality constraints arise, for example, when two reactions are identical but occur in different compartments,[1] or when a reaction is irreversible. For ease of TCMC implementation, we assume that certain reactions in the Schoeberl model that do not depend on any kinetic parameters are characterized by two (dimensionless) rate constants whose values are set equal to zero. In Table S3, we also summarize the estimated kinetic parameter values resulting from TCMC alongside their published values. For clarity, we have rounded the estimated values, although the accompanying SBML file provides the full values.

We should make a note here about units. In the BioModels database (and hence in the software accompanying this document), molecular concentrations are measured in units of $molecules$, which implies rate constants with units of $1/minutes$ or $1/(molecules \times minutes)$ for monomolecular and bimolecular reactions, respectively. In the Main Text, however, we consider a standard approach in which the concentration of molecular species is measured in mol/m^3. Therefore, the rate constants in the Main Text have units of $1/minutes$ or $1/(mol \times minutes)$ for monomolecular and bimolecular reactions, respectively. Conversion between the two cases is straightforward, since molecular numbers can be converted to concentrations by dividing the former by AV, where A is the Avogadro number and V is the system volume. Note that the system volume considered in the BioModels database for the EGF/ERK model is $V = 1p\text{L} = 10^{-15}$ m^3.

[1]The validity of this assumption may be questionable, since kinetic parameters may depend on the compartmental volume. However, the assumption conveniently reduces the complexity of parameter estimation.

Table S1. Reactions that must be removed from the EGF/ERK signaling cascade model in order to obtain a closed subsystem.

**reaction**	**reason for removal**
v5	irreversible reaction
v7	irreversible reaction
v9	irreversible reaction
v13	irreversible reaction
v15	irreversible reaction
v19	GDP turns into GTP with no phosphate source (simplified reaction mechanism)
v21	GDP turns into GTP with no phosphate source (simplified reaction mechanism)
v27	GDP turns into GTP with no phosphate source (simplified reaction mechanism)
v31	GDP turns into GTP with no phosphate source (simplified reaction mechanism)
v36	irreversible reaction
v43	irreversible reaction
v45	irreversible reaction and phosphate is left unbalanced (simplified reaction mechanism)
v47	irreversible reaction and phosphate is left unbalanced (simplified reaction mechanism)
v49	irreversible reaction and phosphate is left unbalanced (simplified reaction mechanism)
v51	irreversible reaction and phosphate is left unbalanced (simplified reaction mechanism)
v53	irreversible reaction and phosphate is left unbalanced (simplified reaction mechanism)
v55	irreversible reaction and phosphate is left unbalanced (simplified reaction mechanism)
v57	irreversible reaction and phosphate is left unbalanced (simplified reaction mechanism)
v59	irreversible reaction and phosphate is left unbalanced (simplified reaction mechanism)
v60	irreversible reaction
v61	irreversible reaction
v62	irreversible reaction
v66	GDP turned into GTP with no phosphate source (simplified reaction mechanism)
v68	GDP turned into GTP with no phosphate source (simplified reaction mechanism)
v74	GDP turned into GTP with no phosphate source (simplified reaction mechanism)
v78	GDP turned into GTP with no phosphate source (simplified reaction mechanism)
v85	irreversible reaction
v87	irreversible reaction and phosphate is left unbalanced (simplified reaction mechanism)
v89	irreversible reaction and phosphate is left unbalanced (simplified reaction mechanism)
v91	irreversible reaction and phosphate is left unbalanced (simplified reaction mechanism)
v93	irreversible reaction and phosphate is left unbalanced (simplified reaction mechanism)
v95	irreversible reaction and phosphate is left unbalanced (simplified reaction mechanism)
v97	irreversible reaction and phosphate is left unbalanced (simplified reaction mechanism)

Table S1. Continued.

reaction	reason for removal
v99	irreversible reaction and phosphate is left unbalanced (simplified reaction mechanism)
v101	irreversible reaction and phosphate is left unbalanced (simplified reaction mechanism)
v107	irreversible reaction
v110	irreversible reaction
v113	irreversible reaction
v116	irreversible reaction
v119	irreversible reaction
v122	irreversible reaction
v125	irreversible reaction

Table S2. Independent reaction cycles in the EGF/ERK signaling cascade model. The entropy production rates σ, associated with the published model, are measured in $\mathrm{J}/(\mathrm{K} \cdot \mathrm{min})$.

Cycle	Reactions	σ
1	v16, v17, v34, v35	-3.58×10^{-14}
2	v16, v17, v24, v25, v32, v33, v34, v37	-7.63×10^{-15}
3	v16, v17, v25, v32, v33, v34, v38, v39	-7.63×10^{-15}
4	v16, v17, v33, v34, v38, v40	$+5.80 \times 10^{-15}$
5	v16, v17, v24, v25, v34, v41	$+9.99 \times 10^{-15}$
6	v22, v23, v24, v25, v32, v69, v70, v71, v72, v79	0
7	v16, v17, v34, v63, v64, v80	0
8	v16, v17, v22, v23, v24, v25, v32, v33, v34, v69, v70, v81	-7.63×10^{-15}
9	v16, v17, v22, v23, v24, v25, v32, v33, v34, v38, v69, v70, v71, v82	-7.63×10^{-15}
10	v16, v17, v34, v71, v72, v83	$+9.99 \times 10^{-15}$
11	v22, v69, v102, v103	0
12	v22, v23, v69, v70, v102, v104	0
13	v16, v17, v63, v64, v102, v105	0
14	v16, v17, v18, v63, v64, v65, v102, v108	0
15	v22, v23, v24, v69, v70, v71, v102, v114	0
16	v22, v23, v24, v25, v69, v70, v71, v72, v102, v117	0
17	v22, v23, v24, v25, v26, v69, v70, v71, v72, v73, v102, v120	0
18	v16, v17, v20, v22, v23, v24, v25, v30, v63, v64, v67, v69, v70, v71, v72, v77, v111, v123	0

Table S3. Published and thermodynamically consistent estimated values of the kinetic parameters associated with the EGF/ERK signaling cascade model. Bold faces indicate non-thermodynamically constrained parameters.

parameter	published value	TCMC value	units
k1	3.00×10^{-3}	2.37×10^{-3}	1/(molecules × min)
kr1	2.28×10^{-1}	1.15×10^{-1}	1/min
k2	1.00×10^{-3}	0.48×10^{-3}	1/(molecules × min)
kr2	6	0.51	1/min
k3	60	31.72	1/min
kr3	6.00×10^{-1}	22.21×10^{-1}	1/min
k4	1.038×10^{-5}	3.05×10^{-5}	1/(molecules × min)
kr4	9.96×10^{-2}	12.31×10^{-2}	1/min
**k5**	0	0	—
**kr5**	0	0	—
k6	3.00×10^{-3}	0.41×10^{-3}	1/min
kr6	3.00×10^{-1}	2.94×10^{-1}	1/min
k7	3×10^{-3}	3.01×10^{-3}	1/min
**kr7**	0	0	1/min
k8	1.00×10^{-4}	5.17×10^{-4}	1/(molecules × min)
kr8	12	0.91	1/min
**k9**	k7	k7	1/min
**kr9**	0	0	1/min
k10	3.25581	3804	1/(molecules × min)
kr10	0.66	172	1/min
**k11**	k2	k2	1/(molecules × min)
**kr11**	kr2	kr2	1/min
**k12**	k3	k3	1/min
**kr12**	kr3	kr3	1/min
k13	130.2	0.46	molecules/min
**kr13**	0	0	1/min
k14	1.00×10^{-4}	6.37×10^{-7}	1/(molecules × min)
kr14	12	197	1/min
k15	60×10^{4}	4.65×10^{4}	1/min
**kr15**	0	0	1/min
k16	1.00×10^{-3}	0.40×10^{-3}	1/(molecules × min)
kr16	16.5	0.45	1/min
k17	1.00×10^{-3}	0.31×10^{-3}	1/(molecules × min)
kr17	3.6	2.52	1/min
k18	1.5×10^{-3}	4.46×10^{-3}	1/(molecules × min)
kr18	78	11.14	1/min
k19	30	350	1/min
kr19	1.00×10^{-5}	0.58×10^{-5}	1/(molecules × min)
k20	2.10×10^{-4}	0.52×10^{-4}	1/(molecules × min)
kr20	24	12.82	1/min

Table S3. Continued.

**parameter**	**published value**	**TCMC value**	**units**
k21<br>kr21	1.38<br>2.2×10^{-5}	0.47<br>1.71×10^{-5}	1/min<br>1/(molecules × min)
k22<br>kr22	2.10×10^{-3}<br>6	0.14×10^{-3}<br>0.62	1/(molecules × min)<br>1/min
k23<br>kr23	360<br>36	420<br>17.39	1/min<br>1/min
k24<br>kr24	1.00×10^{-3}<br>33	7.18×10^{-3}<br>563	1/(molecules × min)<br>1/min
k25<br>kr25	1.00×10^{-3}<br>1.284	0.69×10^{-3}<br>1.22	1/(molecules × min)<br>1/min
**k26**<br>**kr26**	k18<br>kr18	k18<br>kr18	1/(molecules × min)<br>1/min
**k27**<br>**kr27**	k19<br>kr19	k19<br>kr19	1/min<br>1/(molecules × min)
k28<br>kr28	1.00×10^{-4}<br>3.18×10^{-1}	0.098×10^{-4}<br>9.68×10^{-1}	1/(molecules × min)<br>1/min
k29<br>kr29	60<br>7.00×10^{-5}	931<br>10.96×10^{-5}	1/min<br>1/(molecules × min)
**k30**<br>**kr30**	k20<br>kr20	k20<br>kr20	1/(molecules × min)<br>1/min
**k31**<br>**kr31**	k21<br>kr21	k21<br>kr21	1/min<br>1/(molecules × min)
k32<br>kr32	6<br>2.40×10^{-5}	14.19<br>5.55×10^{-5}	1/min<br>1/(molecules × min)
k33<br>kr33	12<br>2.10×10^{-3}	10.96<br>0.017×10^{-3}	1/min<br>1/(molecules × min)
k34<br>kr34	1.8<br>4.50×10^{-4}	0.25<br>1.28×10^{-4}	1/min<br>1/(molecules × min)
k35<br>kr35	0.09<br>4.50×10^{-4}	1.84<br>3.87×10^{-4}	1/min<br>1/(molecules × min)
Km36<br>Vm36	2.00×10^{14}<br>61200	7.72×10^{14}<br>615	molecules<br>molecules/min
k37<br>kr37	18<br>9.00×10^{-5}	29.35<br>0.55×10^{-5}	1/min<br>1/(molecules × min)
**k38**<br>**kr38**	k24<br>kr24	k24<br>kr24	1/(molecules × min)<br>1/min
**k39**<br>**kr39**	k37<br>kr37	k37<br>kr37	1/min<br>1/(molecules × min)
k40<br>kr40	3.00×10^{-3}<br>3.84	0.074×10^{-3}<br>2.75	1/(molecules × min)<br>1/min
k41<br>kr41	3.00×10^{-3}<br>2.574	1.52×10^{-3}<br>44.60	1/(molecules × min)<br>1/min

Table S3. Continued.

**parameter**	**published value**	**TCMC value**	**units**
k42	7.10×10^{-3}	9.69×10^{-3}	1/(molecules × min)
kr42	12	1.87	1/min
k43	60	51.61	1/min
**kr43**	0	0	1/(molecules × min)
k44	1.11×10^{-3}	1.41×10^{-3}	1/(molecules × min)
kr44	1.0998	0.599	1/min
k45	210	6340	1/min
**kr45**	0	0	1/(molecules × min)
**k46**	k44	k44	1/(molecules × min)
**kr46**	kr44	kr44	1/min
k47	174	1632	1/min
**kr47**	0	0	1/(molecules × min)
k48	1.43×10^{-3}	0.69×10^{-3}	1/(molecules × min)
kr48	48	1489	1/min
k49	3.48	10.73	1/min
**kr49**	0	0	1/(molecules × min)
k50	2.50×10^{-5}	54.64×10^{-5}	1/(molecules × min)
kr50	30	9.95	1/min
**k51**	k49	k49	1/min
**kr51**	0	0	1/(molecules × min)
k52	5.34×10^{-3}	3.83×10^{-3}	1/(molecules × min)
kr52	1.98	19.85	1/min
k53	960	62182	1/min
**kr53**	0	0	1/(molecules × min)
**k54**	k52	k52	1/(molecules × min)
**kr54**	kr52	kr52	1/min
k55	342	1120	1/min
**kr55**	0	0	1/(molecules × min)
k56	1.45×10^{-3}	4.70×10^{-3}	1/(molecules × min)
kr56	36	1.23	1/min
k57	16.20	19.75	1/min
**kr57**	0	0	1/(molecules × min)
k58	5.00×10^{-4}	1.71×10^{-4}	1/(molecules × min)
kr58	30	0.114	1/min
k59	18	6.41	1/min
**kr59**	0	0	1/(molecules × min)
k60	4.00×10^{-2}	8.69×10^{-2}	1/min
**kr60**	0	0	1/min
k61	10.02×10^{-3}	6.50×10^{-3}	1/min
**kr61**	0	0	1/min
**k62**	k60	k60	1/min
**kr62**	0	0	1/min

Table S3. Continued.

parameter	published value	TCMC value	units
**k63**	k16	k16	1/(molecules × min)
**kr63**	kr16	kr16	1/min
**k64**	k17	k17	1/(molecules × min)
**kr64**	kr17	kr17	1/min
**k65**	k18	k18	1/(molecules × min)
**kr65**	kr18	kr18	1/min
**k66**	k19	k19	1/min
**kr66**	kr19	kr19	1/(molecules × min)
**k67**	k20	k20	1/(molecules × min)
**kr67**	kr20	kr20	1/min
**k68**	k21	k21	1/min
**kr68**	kr21	kr21	1/(molecules × min)
**k69**	k22	k22	1/(molecules × min)
**kr69**	kr22	kr22	1/min
**k70**	k23	k23	1/min
**kr70**	kr23	kr23	1/min
**k71**	k24	k24	1/(molecules × min)
**kr71**	kr24	kr24	1/min
**k72**	k25	k25	1/(molecules × min)
**kr72**	kr25	kr25	1/min
**k73**	k18	k18	1/(molecules × min)
**kr73**	kr18	kr18	1/min
**k74**	k19	k19	1/min
**kr74**	kr19	kr19	1/(molecules × min)
**k75**	k28	k28	1/(molecules × min)
**kr75**	kr28	kr28	1/min
**k76**	k29	k29	1/min
**kr76**	kr29	kr29	1/(molecules × min)
**k77**	k20	k20	1/(molecules × min)
**kr77**	kr20	kr20	1/min
**k78**	k21	k21	1/min
**kr78**	kr21	kr21	1/(molecules × min)
**k79**	k32	k32	1/min
**kr79**	kr32	kr32	1/(molecules × min)
**k80**	k34	k34	1/min
**kr80**	kr34	kr34	1/(molecules × min)
**k81**	k37	k37	1/min
**kr81**	kr37	kr37	1/(molecules × min)
**k82**	k37	k37	1/min
**kr82**	kr37	kr37	1/(molecules × min)
**k83**	k41	k41	1/(molecules × min)
**kr83**	kr41	kr41	1/min

Table S3. Continued.

parameter	published value	TCMC value	units
**k84**	k42	k42	1/(molecules × min)
**kr84**	kr42	kr42	1/min
**k85**	k43	k43	1/min
**kr85**	0	0	1/(molecules × min)
**k86**	k44	k44	1/(molecules × min)
**kr86**	kr44	kr44	1/min
**k87**	k45	k45	1/min
**kr87**	0	0	1/(molecules × min)
**k88**	k44	k44	1/(molecules × min)
**kr88**	kr44	kr44	1/min
**k89**	k47	k47	1/min
**kr89**	0	0	1/(molecules × min)
**k90**	k48	k48	1/(molecules × min)
**kr90**	kr48	kr48	1/min
**k91**	k49	k49	1/min
**kr91**	0	0	1/(molecules × min)
**k92**	k50	k50	1/(molecules × min)
**kr92**	kr50	kr50	1/min
**k93**	k49	k49	1/min
**kr93**	0	0	1/(molecules × min)
**k94**	k52	k52	1/(molecules × min)
**kr94**	kr52	kr52	1/min
**k95**	k53	k53	1/min
**kr95**	0	0	1/(molecules × min)
**k96**	k52	k52	1/(molecules × min)
**kr96**	kr52	kr52	1/min
**k97**	k53	k53	1/min
**kr97**	0	0	1/(molecules × min)
**k98**	k56	k56	1/(molecules × min)
**kr98**	kr56	kr56	1/min
**k99**	k57	k57	1/min
**kr99**	0	0	1/(molecules × min)
**k100**	k58	k58	1/(molecules × min)
**kr100**	kr58	kr58	1/min
**k101**	k59	k59	1/min
**kr101**	0	0	1/(molecules × min)
**k102**	k6	k6	1/min
**kr102**	kr6	kr6	1/min
**k103**	k6	k6	1/min
**kr103**	kr6	kr6	1/min
**k104**	k6	k6	1/min
**kr104**	kr6	kr6	1/min

Table S3. Continued.

parameter	published value	TCMC value	units
**k105**	k6	k6	1/min
**kr105**	kr6	kr6	1/min
**k106**	k4	k4	1/(molecules × min)
**kr106**	kr4	kr4	1/min
**k107**	0	0	—
**kr107**	0	0	—
**k108**	k6	k6	1/min
**kr108**	kr6	kr6	1/min
**k109**	k4	k4	1/(molecules × min)
**kr109**	kr4	kr4	1/min
**k110**	0	0	—
**kr110**	0	0	—
**k111**	k6	k6	1/min
**kr111**	kr6	kr6	1/min
**k112**	k4	k4	1/(molecules × min)
**kr112**	kr4	kr4	1/min
**k113**	0	0	—
**kr113**	0	0	—
**k114**	k6	k6	1/min
**kr114**	kr6	kr6	1/min
**k115**	k4	k4	1/(molecules × min)
**kr115**	kr4	kr4	1/min
**k116**	0	0	—
**kr116**	0	0	—
**k117**	k6	k6	1/min
**kr117**	kr6	kr6	1/min
**k118**	k4	k4	1/(molecules × min)
**kr118**	kr4	kr4	1/min
**k119**	0	0	—
**kr119**	0	0	—
**k120**	k6	k6	1/min
**kr120**	kr6	kr6	1/min
**k121**	k4	k4	1/(molecules × min)
**kr121**	kr4	kr4	1/min
**k122**	0	0	—
**kr122**	0	0	—
**k123**	k6	k6	1/min
**kr123**	kr6	kr6	1/min
**k124**	k4	k4	1/(molecules × min)
**kr124**	kr4	kr4	1/min
**k125**	0	0	—
**kr125**	0	0	—

Simulated Annealing

Simulated annealing (SA) algorithms come in many varieties; for an introduction, see [6]. Here, we present the specific algorithm we use in the EGF/ERK signaling cascade example. This algorithm employs a geometrically decaying annealing schedule and uses a zero-mean multivariate Gaussian proposal distribution.

Initialization

1. Set an initial value λ for the annealing schedule and a value for its decay rate $0 < \delta < 1$. We use $\lambda = 2 \times 10^{12}$ and $\delta = 0.97$.
2. Set an initial value for the standard deviation φ of the Gaussian proposal distribution and a value for its decay rate $0 < \gamma < 1$. We use $\varphi = 1.5$ and $\gamma = 0.996$.
3. Set the total allowable number S of cost function evaluations and the number of iterations U for each annealing update. We use $S = 100{,}000$ and $U = 50$.
4. Form matrix $\mathbb{A}$ and vector $\boldsymbol{c}$ and find a particular solution $\boldsymbol{\kappa}_0$ of $\mathbb{A}\boldsymbol{\kappa}_0 = \boldsymbol{c}$ that is closest, in the least-squares sense, among all other solutions to the published parameter values. Set $\boldsymbol{v}(1) = 0$, and calculate the initial cost $c(1) = C_0(\boldsymbol{v}(1) \mid \boldsymbol{y}) = C(\boldsymbol{\kappa}_0 \mid \boldsymbol{y})$.

Iteration

For $s = 1, 2, \ldots, S$:

5. If U iterations have passed since the last update of the annealing schedule, set $\lambda = \delta\lambda$ and $\varphi = \gamma\varphi$.
6. Given the current value $\boldsymbol{v}(s)$, randomly draw a new proposed value $\boldsymbol{v}'(s)$ from a multivariate Gaussian distribution with mean $\boldsymbol{v}(s)$ and covariance matrix $\varphi^2\mathbb{I}$, where $\mathbb{I}$ is the identity matrix.
7. Calculate the cost $c'(s) = C_0(\boldsymbol{v}'(s) \mid \boldsymbol{y}) = C(\boldsymbol{\kappa}_0 + \mathbb{B}\boldsymbol{v}'(s) \mid \boldsymbol{y})$.
8. If $c'(s) < c(s)$, then set $\boldsymbol{v}(s+1) = \boldsymbol{v}'(s)$ and $c(s+1) = c'(s)$. Otherwise, calculate $p = \exp\{-[c'(s) - c(s)]/\lambda\}$ and set $\boldsymbol{v}(s+1) = \boldsymbol{v}'(s)$, $c(s+1) = c'(s)$ with probability p, or $\boldsymbol{v}(s+1) = \boldsymbol{v}(s)$, $c(s+1) = c(s)$ with probability $1 - p$.

Estimation

9. Choose as the final estimated value $\widehat{\boldsymbol{v}}$ of $\boldsymbol{v}$ the point associated with the minimum cost among all calculated cost values $\{c(1), c(2), \ldots, c(S)\}$, and set $\widehat{\boldsymbol{\kappa}} = \boldsymbol{\kappa}_0 + \mathbb{B}\widehat{\boldsymbol{v}}$.

There are many possible choices for the annealing schedule and the proposal distribution. However, the ones used in this paper (namely the geometrically decaying annealing schedule and the i.i.d. Gaussian

proposal distribution with geometrically decaying standard deviation) are common choices [6].
A geometrically decaying annealing schedule ensures that, at early iterations, the algorithm is allowed to explore the parameter space even if the cost function increases, whereas, the geometric decay reduces the probability that a proposed point with higher cost than the current estimate will be accepted. Furthermore, as iterations progress and the parameter estimates improve, the proposal distribution we use in this paper provides, with high probability, points that are closer to the current estimate (and, therefore, more likely to be descent estimates themselves). Finally, Step 9 makes sense, since it chooses the best parameter estimate encountered during the SA iterations.

Additional Results

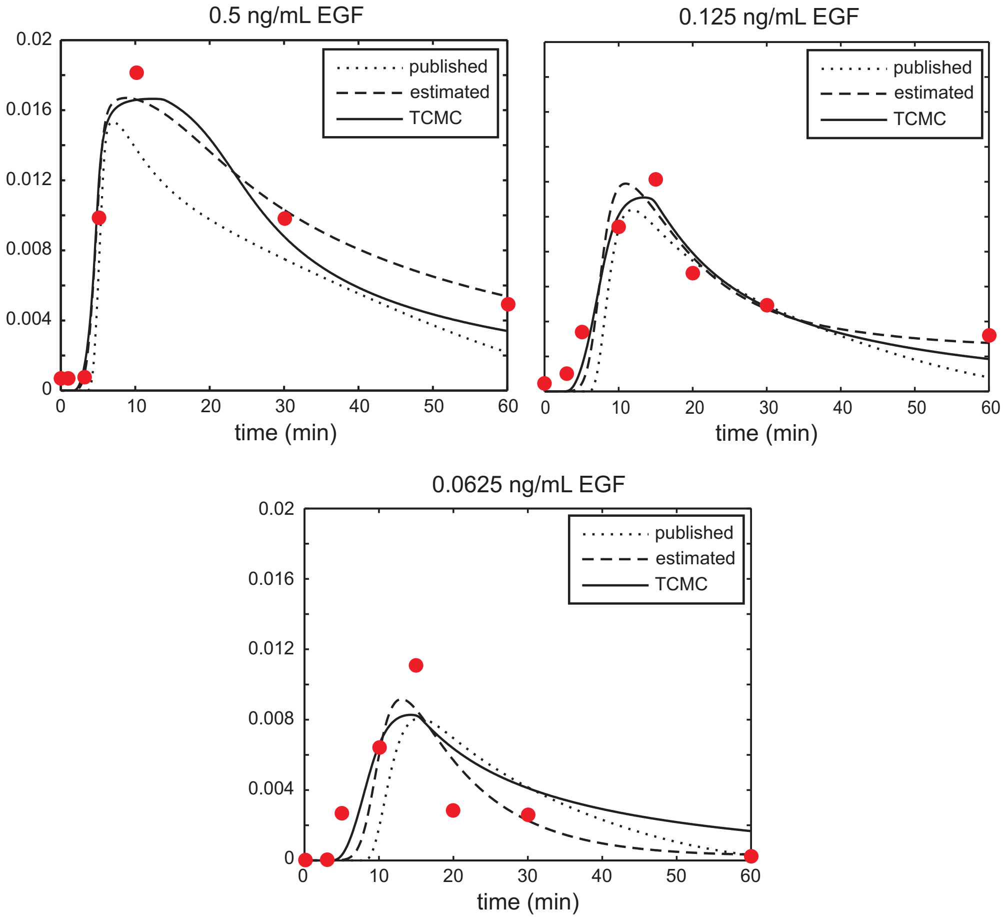

Figure S.1: ERK-PP concentration dynamics, measured in mol/m^3, under three different input EGF concentrations. These dynamics are in addition to the ones depicted in Figure 1 of the Main Text. The red circles indicate densitometric data obtained from Schoeberl *et al.* [4].

References

1. Israel A, Greville TNE: *Generalized Inverses: Theory and Applications*, 2nd ed. New York: Springer-Verlag 2003.

2. Vlad MO, Ross J: **Thermodynamically based constraints for rate coefficients of large biochemical networks**. *WIREs Syst Biol Med* 2009, **1**: 348–358.

3. Qian H, Beard DA: **Thermodynamics of stoichiometric biochemical networks in living systems far from equilibrium**. *Biophys Chem* 2005, **114**(2-3): 213–220.

4. Schoeberl B, Eichler-Jonsson C, Gilles ED, Müller G: **Computational modeling of the dynamics of the MAP kinase cascade activated by surface and internalized EGF receptors**. *Nat Biotechnol* 2002, **20**: 370–375.

5. Li C, Donizelli M, Rodriguez N, Dharuri H, Endler L, Chelliah V, Li L, He E, Henry A, Stefan MI, Snoep JL, Hucka M, Le Novère N, Laibe C: **BioModels database: An enhanced, curated and annotated resource for published quantitative kinetic models**. *BMC Syst Biol* 2010, **4**: 92.

6. Spall JC: *Introduction to Stochastic Search and Optimization: Estimation, Simulation, and Control*. Hoboken: Wiley 2003.
